# Artificial Intelligence-Assisted Selection Strategies in Sheep: Linking Reproductive Traits with Behavioral Indicators

**DOI:** 10.3390/ani15142110

**Published:** 2025-07-17

**Authors:** Ebru Emsen, Muzeyyen Kutluca Korkmaz, Bahadir Baran Odevci

**Affiliations:** 1 Department of Integrative Agriculture, College of Agriculture and Veterinary Medicine, United Arab Emirates University, Al Ain P.O. Box 15551, United Arab Emirates; 2 Department of Animal Science, Faculty of Agriculture, Malatya Turgut Ozal University, 44210 Malatya, Turkey; muzeyyen.korkmaz@ozal.edu.tr; 3 Imona Technologies, ITU Ari Teknokent, 34396 Istanbul, Turkey; bahadir.odevci@gmail.com

**Keywords:** artificial intelligence, behavioral phenotyping, reproductive traits, sheep, selection strategies, machine learning, livestock technology

## Abstract

Reproductive efficiency is a critical determinant of productivity and profitability in sheep farming. Traditional selection methods have largely relied on phenotypic traits and historical reproductive records, which are often limited by subjectivity and delayed feedback. Recent advancements in artificial intelligence (AI), including video tracking, wearable sensors, and machine learning (ML) algorithms, offer new opportunities to identify behavior-based indicators linked to key reproductive traits such as estrus, lambing, and maternal behavior. This review synthesizes the current research on AI-powered behavioral monitoring tools and proposes a conceptual model, ReproBehaviorNet, that maps age- and sex-specific behaviors to biological processes and AI applications, supporting real-time decision-making in both intensive and semi-intensive systems. The integration of accelerometers, GPS systems, and computer vision models enables continuous, non-invasive monitoring, leading to earlier detection of reproductive events and greater breeding precision. However, the implementation of such technologies also presents challenges, including the need for high-quality data, a costly infrastructure, and technical expertise that may limit access for small-scale producers. Despite these barriers, AI-assisted behavioral phenotyping has the potential to improve genetic progress, animal welfare, and sustainability. Interdisciplinary collaboration and responsible innovation are essential to ensure the equitable and effective adoption of these technologies in diverse farming contexts.

## Simple Summary

Sheep farmers aim to breed animals that are healthy, fertile, and able to care for their lambs. Traditionally, they have selected animals based on physical traits and past performance. However, watching animals to identify important behaviors, like readiness to mate or how well a mother cares for her lamb, can be time-consuming and difficult. This study explores how modern technologies such as cameras, movement sensors, and computer systems using artificial intelligence can help farmers make better breeding decisions. These tools can track the behavior of sheep and detect signs like increased activity before mating or how close a mother stays to her lamb. By turning these behaviors into useful information, farmers can select animals more effectively and improve the health and survival of future generations. This new approach also reduces the need for stressful handling of animals and supports more humane farming. Our work shows how technology can be used in a smart and responsible way to help both farmers and animals, leading to more productive and sustainable sheep farming.

## 1. Introduction

The reproductive performance of sheep is a crucial determinant of overall production efficiency and economic viability in sheep farming systems [1]. Traditionally, selection strategies for enhancing reproductive traits in sheep have relied on direct measurements such as litter size, lambing interval, and ewe fertility, often augmented by pedigree information and, more recently, genomic data. These traditional approaches, while valuable, are often limited by the time-consuming and labor-intensive nature of data collection, as well as the potential for inaccuracies due to subjective assessments or environmental influences. In order to further bolster sheep farming, advanced technological applications have been developed, which enhance the ability to control, monitor, and manage farm animal activities. These new technologies have been recognized as valuable tools for monitoring and managing various aspects of sheep production, particularly in extensive, pasture-based systems [2]. Among these, artificial intelligence (AI) stands out as a broad field of computer science that includes multiple subfields, such as machine learning (ML), deep learning (DL), and large language models (LLMs). ML refers to algorithms that learn from data to make predictions or decisions without being explicitly programmed. DL, a subset of ML, uses artificial neural networks with multiple layers to recognize patterns in complex datasets. LLMs are designed to process and generate human language. In livestock research, AI applications primarily rely on ML and DL to monitor animal behavior, predict reproductive performance, and support precise decision-making. The application of technologies such as video tracking, wearable sensors, and ML algorithms can deepen our understanding of animal behavior and improve overall animal welfare and health, contributing to the sustainability of sheep farming. Emerging technologies in the realm of AI and behavioral monitoring offer promising tools to complement and enhance these traditional selection methods by providing objective, real-time insights into behavioral indicators that are intrinsically linked to reproductive success [3]. These technologies not only improve automation and decision-making in livestock systems, but also create new techniques to observe areas such as animal health, management, nutrition, and welfare [3,4]. The review emphasizes the potential of integrating AI-assisted behavioral phenotyping into existing farm management systems. This integration can enable real-time decision support, leading to improved flock-level reproductive performance. By focusing on non-invasive, technology-driven selection strategies, this review underscores the importance of interdisciplinary collaboration to advance breeding programs while enhancing animal welfare and sustainability. The application of AI and behavioral monitoring technologies can provide valuable insights into key reproductive traits, allowing breeders to make more informed decisions and drive genetic improvements that promote efficient and sustainable sheep production.

## 2. Integrating Behavioral Indicators with Reproductive Traits

### 2.1. Female Behavioral Indicators

Behavioral attributes, including activity patterns, social dynamics, and reactions to environmental factors, offer significant insights into an animal’s overall well-being and its capacity to adjust to varying management practices; these factors can indirectly affect reproductive success [5,6,7,8]. These behavioral attributes not only reflect general well-being, but also signal physiological readiness for reproduction. Establishing clear links between observable behaviors and internal reproductive states is essential for improving detection and selection strategies. Therefore, it is important to consider how specific behaviors correspond with key reproductive stages. Reproductive performance in sheep can be categorized into key stages, including the onset of puberty, marked by the display of estrous behavior; the age at first successful pregnancy, accompanied by effective mating; lambing rates, influenced by careful environmental management during gestation with a focus on mitigating stress; and postpartum maternal ability, characterized by the ewe’s proficiency in caring for her offspring. These reproductive stages are commonly associated with performance indicators such as conception rate, litter size, and lambing interval, though the current review focuses on their behavioral manifestations. Understanding the behavioral and physiological traits exhibited during different reproductive stages is essential for optimizing reproductive management and enhancing breeding outcomes [1].

The reproductive performance of a sheep flock does not only encompass mature animals; it also requires significant attention to be given to young animals during the pubertal transition. Accurate evaluation of the onset of puberty is crucial, as early sexual maturity is one of the most important selection criteria for meat and dairy sheep enterprises [9]. The expression of behavioral indicators associated with the onset of puberty in ewe lambs can be subtle and asynchronous, especially when contrasted with male lambs, which tend to reach puberty at an earlier age; however, this pattern is influenced by factors such as breed, nutrition, and environmental conditions, which can modulate the timing of sexual maturation in both sexes [10]. These subtle differences can make it challenging to accurately determine the optimal time for breeding interventions, leading to inefficiencies in reproductive management and potentially lower conception rates. Additionally, the asynchronous nature of puberty onset within a group of ewe lambs further complicates management decisions, as not all individuals will be ready for breeding at the same time [11]. This variability necessitates careful monitoring and individualized management strategies to ensure that each ewe lamb is bred at the appropriate time, maximizing reproductive success and minimizing the risk of complications during pregnancy and lambing [12]. Therefore, integrating behavioral data with physiological markers can provide a more comprehensive assessment of puberty onset in ewe lambs, which allows for more targeted and effective breeding strategies, leading to more productive selection in sheep farms, where both behavioral and reproductive data should be integrated into farm management and monitoring systems [13].

Estrus behavior in sheep, characterized by a range of changes including increased activity levels, frequent vocalizations, and heightened interactions with male counterparts, can be accurately detected and quantified through advanced technologies such as video tracking systems [14,15], accelerometers [16], sensors [17], and Global Navigation Satellite System (GNSS) tracking devices [18], as well as by using estrus detection patches or rams equipped with marking harnesses [19] for estrus detection in synchronized ewes. These technologies offer a non-invasive means of continuously monitoring sheep behavior, providing valuable insights into estrus patterns and facilitating more precise management of breeding programs. These data-driven strategies enhance the accuracy and efficiency of estrus detection, which is crucial for optimizing breeding outcomes, including precise timing for artificial insemination procedures in sheep, and for minimizing the need for labor-intensive manual observations. The integration of modern technologies with traditional management practices can significantly improve the efficiency and effectiveness of sheep breeding programs by improving productivity, maximizing reproductive success, and enhancing animal welfare.

### 2.2. Male Behavioral Indicators

Although much of the focus in reproductive behavior studies has traditionally been placed on females, male animals, particularly rams, are often overlooked despite their substantial contribution to reproductive outcomes [13]. Rams tend to exhibit more distinct and observable behavioral patterns than ewes, especially during the breeding season, including increased mounting activity, the flehmen response, and persistent courtship behaviors. These behaviors are not only indicators of sexual motivation, but are also closely linked to semen quality and mating success [20]. Moreover, temperament traits such as emotional reactivity, docility, or tameness have been shown to influence reproductive capacity, either directly through hormonal pathways such as testosterone and cortisol or indirectly by modifying social interactions and stress responses [21]. Therefore, understanding and quantifying behavioral indicators in rams is essential for improving reproductive efficiency, especially in both natural mating and assisted reproductive systems.

Social hierarchy has emerged as a key behavioral indicator that influences reproductive performance in rams, particularly by shaping behavioral interactions and access to reproductive opportunities in group-housed settings. In a recent study on Dorper rams managed under an intensive system, high-social-ranked (HSR) males exhibited significantly more appetitive and consummatory sexual behaviors, including increased approaches, anogenital sniffing, and mounting with ejaculation, compared to low-social-ranked (LSR) rams [22]. Interestingly, these differences were not associated with live weight but were strongly correlated with higher body condition scores (BCS), suggesting a potential link between dominance-driven access to resources and improved reproductive traits; however, this association is correlational and may be influenced by other confounding factors such as age and genetic background, which warrant further investigation. HSR rams also showed reduced latency to ejaculation and greater ejaculate volume, indicating better sexual readiness and semen quality. Additionally, HSR rams demonstrated lower leukocyte counts, which may suggest altered immunological status; however, this observation should be interpreted cautiously, as leukocyte reductions could also indicate immunosuppression in the absence of additional immune function markers. These findings reinforce the notion that social dominance and related behavioral traits are robust predictors of reproductive success in rams, particularly during out-of-season breeding when sexual activity may otherwise be suppressed. Therefore, integrating social dynamics into selection strategies has the potential to identify and prioritize rams with superior reproductive capabilities, increasing the efficiency and productivity of sheep breeding operations.

Environmental factors, such as increased stress levels in rams, which can be detected through behavioral indicators like reduced activity, altered vocalizations, or changes in social interactions, may lead to a reduction in semen quality and libido, thereby compromising their reproductive potential. This stress can be acute or chronic, and is often associated with physiological markers such as elevated cortisol levels and leukocytospermia, as shown in recent studies [23,24]. Rams subjected to stress-inducing social rearrangements show increased serum cortisol levels alongside cytological alterations in semen [25].

Mating is another critical stage that exhibits many unexplained dynamics in terms of pair formation. It has been observed that certain breeds of rams or ewes exhibit preferences for mating partners. This could be attributed to various factors, including breed-specific behaviors, pheromonal signals, or other cues that influence mate selection. Rams’ libido and serving capacity can be estimated by selected measures of sexual libido when rams are exposed to estrous ewes under conditions that prevent copulation [26].

Sexual behavior in rams not only reflects their libido and reproductive capacity, but also involves complex patterns of mate preference that influence flock fertility. While dominant rams typically gain more mating opportunities through competition, studies suggest that this does not always align with female preference or reproductive efficiency. For instance, Díaz et al. [27] demonstrated that estrous ewes consistently preferred to interact with and be mated by subordinate rather than dominant rams when male activity was restricted, possibly to avoid aggressive courtship and ensure greater genetic diversity in the offspring. Complementing this finding, Abecia et al. [28] showed that Rasa Aragonesa rams exhibited non-random mating behavior, spending significantly more time courting specific ewes, indicating individual preferences that could affect mating outcomes. These behavioral dynamics highlight the need to consider both male and female mating preferences in breeding management, as individual biases and social hierarchies can significantly influence mating success and reproductive performance. Understanding these complex dynamics can provide valuable insights into optimizing mating strategies and improving reproductive outcomes in sheep breeding programs.

Dominance hierarchies among rams play a crucial role in their reproductive success, influencing their access to ewes and, consequently, their mating opportunities [29]. In a study by Ungerfeld and González-Pensado [30], dominant males maintained stable sexual behavior regardless of the presence of rival rams, whereas subordinate rams exhibited a marked reduction in mating activity when competition was introduced. This suggests that dominant rams are not only more assertive in social interactions, but also less susceptible to behavioral inhibition, allowing them to sustain higher mating success. These findings emphasize the importance of social rank as a key behavioral indicator linked to reproductive efficiency in rams. By assessing behavioral traits such as aggressiveness and social interactions, farmers can identify the most dominant and reproductively capable rams within their flock.

Additionally, age is a factor that influences the selection of mates by male animals. In group mating scenarios, some females may be overlooked and not mated, with a preference shown by the rams. Moreover, aggression during mating can lead to competition among rams, which can even be fatal. Recent studies have indicated that females prefer calm rams over aggressive ones. Therefore, mating should be monitored in a non-subjective and non-disturbing manner to allow the animals to exhibit their innate behaviors [22]. Careful observation and management of ram behavior can contribute to more effective breeding strategies and improved reproductive outcomes in sheep husbandry.

### 2.3. Maternal Behavior Indicators

Reproductive success is largely determined by the ewe’s postpartum maternal ability to raise healthy, thriving lambs. Key metrics of productivity include the total number of lambs weaned per ewe as well as the birth weight of the offspring, both of which are closely linked to the mother’s caregiving proficiency. Behavioral indicators related to the mother–offspring relationship, including measures such as nursing frequency, maternal care behaviors, and lamb activity levels, can be utilized to assess the quality of this bond, which is critical for lamb survival and growth [31]. A ewe’s ability to bond with her lambs, provide adequate care, safeguard them from danger, and respond to their needs are all pivotal elements of maternal quality. Maternal behavior, which includes the ewe’s capacity to form a strong attachment to her offspring, offer sufficient care and protection, and be attentive to their needs, is a crucial aspect of maternal quality that can significantly impact lamb survival and productivity [32]. The “maternal behavior score” is often used to quantify ewe behavior under field conditions. It assesses the ewe’s flight distance when lambs are handled shortly after birth, with higher scores indicating a ewe that remains closer to her lamb(s) [33]. Behavioral studies of sheep have demonstrated that inadequate maternal care by the ewe right after birth can result in decreases in lamb survival rates and the overall productivity of the ewe’s flock. These behaviors include grooming, low-pitched bleats, showing no aggression towards the lamb, not deserting the lamb, cooperating with the lambs’ suckling attempts, recognizing the lamb, and maintaining close contact. These are commonly assessed through the Maternal Behavior Score (MBS), which is based on direct observation protocols shortly after lambing, particularly measuring the ewe’s flight distance when the lamb is handled. While individual behaviors are observed qualitatively, the scoring system aggregates them into a composite measure, although the relative weight of each behavior may vary across different studies or breeds [34]. Using a maternal behavior score based on the ewe’s flight distance when lambs are handled for the first time can help quantify maternal behavior. Genetic evaluations in Australian sheep populations have demonstrated that maternal behavior and temperament at lambing are positively associated with lamb survival, suggesting a correlative and potentially causal relationship, especially as these traits have been successfully incorporated into selection programs and shown to improve survival outcomes over time [35]. Notably, advanced analytical tools, particularly ML algorithms, have demonstrated effectiveness in pinpointing crucial predictors of maternal success in sheep, with parturition duration and lamb birth weight emerging as significant determinants of overall outcomes [32].

Accurately predicting the onset of lambing is essential for ensuring the survival of offspring, as well as implementing appropriate management interventions. Lamb losses, especially those caused by dystocia, represent a major economic and welfare issue, with up to 80% of neonatal deaths occurring within a few days after birth due to mismothering, starvation, or exposure. Monitoring the behavior of sheep in especially extensive farming systems is challenging due to large paddock sizes, high animal numbers, and labor costs. Wearable sensor technologies, such as triaxial accelerometers and temperature loggers, now provide an opportunity to continuously track pre-lambing and lambing behaviors in ewes without the need for constant human observation. These tools have been successfully applied in previous studies to detect posture changes, restlessness, and other behavioral shifts indicative of the onset of parturition with high temporal resolution and accuracy. Traditional methods such as GPS tracking could only detect the day of lambing, not the precise time. Therefore, there is a pressing need for accurate, automated tools to predict lambing time, enabling timely intervention [36]. In the period preceding lambing, ewes exhibit distinct behavioral changes that can be identified through accelerometer data and visual observation. These pre-labor behaviors include increased restlessness, frequent transitions between standing and lying, and pawing the ground as they prepare a nesting area. Ewes may also bend their necks backward or laterally, often interpreted as a response to internal discomfort or the onset of uterine contractions. These signs reflect the early physiological and behavioral responses leading up to labor. Although these behaviors can overlap with those observed during labor itself, their consistent appearance prior to parturition makes them valuable indicators for early detection systems. In a recent study, such behaviors were grouped under the ‘pre-labour’ category and analyzed using deep learning models—specifically Long Short-Term Memory (LSTM) neural networks—applied to halter-mounted accelerometer data. The models demonstrated a promising performance, achieving up to 86.3% accuracy and recall values of 0.88 for labor and 0.94 for licking behaviors, thereby supporting their utility in detecting lambing events and prolonged labor phases [37]. These behaviors provide critical insights for both prediction and real-time monitoring. While pre-labor behaviors are primarily used to predict the onset of lambing, continuous tracking of behavioral shifts during active labor enables real-time management and intervention to support ewe and lamb welfare. Therefore, proactive monitoring and timely intervention in cases of difficult births are crucial for fostering strong maternal bonds and maximizing lamb survival rates, thereby contributing to the economic viability of sheep farming enterprises.

The implementation of sophisticated monitoring technologies and advanced analytical methods allows for a deeper exploration of the complexities of maternal care in sheep, enabling the identification and selection of individuals exhibiting superior maternal traits. By selecting ewes that consistently demonstrate strong maternal behaviors, sheep breeders can improve lamb survival rates, optimize growth trajectories, and increase overall productivity. Ultimately, this contributes to a more resilient and economically viable sheep production model, characterized by reduced lamb mortality, enhanced resource utilization, and improved profitability for producers.

## 3. Integration of AI-Powered Tools and Technologies in Behavioral Monitoring of Sheep Reproduction

The digitization of farm production processes allows the monitoring of production performance, animal health, and overall well-being [38]. The integration of AI-powered tools and advanced behavioral monitoring technologies in sheep reproduction denotes a fundamental shift in traditional selection methodologies. This paradigm shift harnesses the power of emerging technologies, such as wearable sensors, video tracking, and ML algorithms, to provide more objective, real-time insights into behavioral indicators that are intrinsically linked to reproductive success. By complementing and enhancing conventional approaches, these innovative techniques offer breeders valuable opportunities to make more informed decisions, drive genetic improvements, and promote efficient and sustainable sheep production [2,39].

In their systematic review of on-animal sensor applications in sheep research, Fogarty et al. [40] analyzed 82 independent experiments conducted across six continents, covering a wide range of climatic conditions including temperate, arid, and cold environments. Most studies focused on the behavioral monitoring of sheep, followed by sensor validation, environmental management, and health-related applications (Table 1). The majority of experiments were conducted using grazing systems, reflecting the practical relevance of extensive production environments. Sensor deployments were typically short-term, with devices attached, removed, and reused across multiple intervals mainly due to battery limitations and the need for high-resolution data. In shorter experiments, sensors were programmed to record continuously or in intervals of <1 min, while longer deployments used extended intervals of 30 to 60 min to optimize energy consumption. Among the sensors, GPS devices were the most used for tracking animal movement and spatial distribution. Motion sensors, such as accelerometers and inertial measurement units (IMUs), were frequently employed to capture activity, posture, and locomotion, with specific reproductive behavior applications summarized in Table 1. Heart rate monitors were utilized primarily for assessing stress and welfare, whereas jaw and bite sensors were applied to detect feeding behaviors (see Table 1), which are associated with nutritional status and may indirectly reflect reproductive readiness, particularly around mating and lambing periods. Contact loggers supported the study of social interactions, and other sensors (e.g., estrus, temperature, or respiration) targeted specific physiological functions. Attachment methods varied, but collars, harnesses, and leg bands were the most frequently used. As sensor technology has advanced, research has shifted from proof-of-concept validation studies toward more complex, integrative monitoring approaches, underscoring the growing role of precision livestock technologies in sheep production systems.

The implementation of AI and ML techniques offers powerful tools for analyzing complex datasets generated from reproductive and behavioral data, enabling more accurate prediction of breeding values and facilitating optimized selection decisions [59]. AI models can identify subtle patterns and correlations between behavioral indicators and reproductive traits that may not be apparent through traditional statistical methods [60]. One key aspect of AI-assisted selection is the use of video tracking and analysis to monitor sheep behavior in various settings [14]. These video tracking and analysis systems can be designed to automatically identify and record specific behaviors in sheep, such as mounting, sniffing, and tail wagging, which are indicative of estrus. Remote monitoring systems also offer the ability to detect patterns of activity related to parturition, including active behaviors like continuous lying and standing, licking of the lamb, and pushing associated with active contractions [61]. The utilization of wearable sensors, such as accelerometers, gyroscopes, and GPS devices, is also essential for tracking and recording various aspects of sheep behavior, including activity levels, movement patterns, and social interactions within the flock. The data collected from these video tracking and wearable sensor systems can provide valuable insights into the behavioral indicators associated with important reproductive traits in sheep [31,36].

Machine learning (ML), a subset of AI, plays a critical role in analyzing the vast amounts of data generated by video tracking and wearable sensors. These algorithms can detect nuanced patterns and associations between behavioral indicators and reproductive performance outcomes, enabling more precise forecasting of estrus [19], lambing ease [62], and maternal behavior [32], as demonstrated in studies on dairy cattle [63,64,65]. By focusing on non-invasive, technology-driven selection strategies, breeders can enhance animal welfare and sustainability, which are increasingly important considerations in modern sheep farming [2]. AI systems excel at processing large-scale datasets, swiftly identifying patterns, and minimizing analytical subjectivity, thereby outperforming human capabilities in specific contexts [60]. Precision livestock farming uses innovative technologies to monitor animals by continuously collecting real-time data, employing sensors and advanced algorithms to aid farmers’ decision-making [60]. Data analytics are essential for effectively evaluating farm performance, facilitating more precise estimates, and refining forecasts and strategies. By combining sensors, cameras, and production data in a cohesive infrastructure, livestock producers can generate predictions that allow them to anticipate detrimental factors that influence animal health and well-being, as well as overall economic sustainability [66]. The integration of behavioral data, captured through non-invasive methods such as video analysis and wearable sensors, offers a novel dimension to selection decisions, allowing breeders to identify animals with superior reproductive fitness based on their natural behaviors.

The integration of AI-based technologies into sheep reproductive management has seen substantial progress with the rise of non-invasive sensing systems. In this context, the review by Wang et al. [67], which synthesizes findings from over 300 peer-reviewed studies published between 2003 and 2024, offers a broad and timely overview of deep learning applications in precision sheep farming. Their analysis categorizes sensor use into four key domains: facial recognition, body metrics estimation, behavioral monitoring, and physiological assessment. The study emphasizes how models such as YOLOv5 and LSTM architectures have achieved high performance in detecting reproductive behaviors, such as estrus (mean Average Precision, mAP > 99%) and lambing signs, based on labeled datasets collected using RGB and thermal cameras under commercial farm conditions, where estrus and parturition-related behaviors were annotated, enabling real-time decision-making in practical field environments. Particularly relevant to our review, their findings highlight the effectiveness of integrating multi-sensor data (e.g., thermal imaging, accelerometry, and video-based monitoring) for parturition detection and maternal behavior analysis. Furthermore, their coverage of facial expression recognition, achieving up to 96.1% accuracy in detecting pain and fear, adds a novel welfare dimension that could potentially serve as an indirect selection trait linked to reproductive success. As outlined in Table 2 of their study, the shift toward minimally intrusive technologies, such as RGB/thermal cameras and wearable sensors, provides scalable, continuous monitoring without disrupting natural behaviors, making them ideal for use in AI-assisted selection strategies. These insights support the integration of behavioral indicators such as grooming, mounting, lying, or isolation into reproductive trait phenotyping, reinforcing the value of precise behavioral data in sheep genetic improvement programs.

## 4. Challenges and Considerations

Selection strategies in sheep breeding have traditionally focused on phenotypic traits, particularly those associated with production, such as body weight and fleece production [68]. However, reproductive efficiency is a crucial factor influencing the profitability and sustainability of sheep farming. Maximizing genetic progress through selection requires the development of effective selection indices and accurate estimates of genetic and non-genetic parameters. Reproductive performance in sheep is influenced by a complex interplay between the animal’s inherent genetic traits and various environmental factors, such as feeding regimes, management practices, health status, welfare conditions, and seasonal influences [69]. Incorporating behavioral data into traditional selection criteria offers a promising avenue to enhance the accuracy and effectiveness of breeding programs [70]. Contemporary breeding goals increasingly emphasize a more holistic approach, integrating both production and welfare traits to ensure long-term sustainability. Analyzing behavioral indicators, which reflect an animal’s adaptation to its environment and social interactions, could provide additional valuable insights into reproductive potential.

The combination of AI and sensor technologies is facilitating a shift in the livestock export sector by improving animal welfare, enhancing operational efficiency, and expanding market access [65]. The adoption of AI-assisted selection strategies in sheep breeding holds immense promise for accelerating genetic improvement, enhancing reproductive efficiency, and promoting sustainable production practices. AI allows the entry of simple data from farm records, monitors farm activities, and analyzes economic performance, ultimately improving animal health [71]. By leveraging the power of AI to integrate reproductive traits with behavioral indicators, breeders can make more informed selection decisions, leading to healthier, more productive, and more resilient flocks.

Despite the potential advantages, a range of challenges and considerations must be addressed to successfully integrate AI-assisted selection strategies into sheep breeding programs. Implementing these strategies within sheep breeding programs necessitates a multifaceted approach that accounts for the specific needs and resources of diverse farming systems. Drawing from the insights gained from cattle farming, the need for tailored AI algorithms and training programs extends to sheep breeding as well. As observed in cattle, the placement and type of wearable sensors may differ based on the size, breed, and behavior of the cow. This highlights the necessity of customizing AI algorithms to account for individual variations between cows [65]. Similarly, in sheep farming, breed-specific behaviors and physiological differences would necessitate the adaptation of AI-driven tools and training programs. Training programs and educational resources are needed to equip farmers and breeders with the skills to effectively utilize AI-driven tools and interpret the resulting data.

Furthermore, the development of standardized data collection protocols and data-sharing platforms is essential to facilitate the creation of large, high-quality datasets that can be used to train and validate AI models across diverse populations of sheep. Data security and privacy are major concerns, as is the technological infrastructure needed, which can be complex [72]. Ensuring data quality and reliability is crucial, as the accuracy of AI models depends on the integrity of the input data. The “black box” nature of some AI algorithms can make it difficult to interpret the underlying biological mechanisms driving the predictions, which can hinder the acceptance and adoption of these technologies by breeders. As Knight [73] noted, without being able to reasonably explain the reasoning behind each AI prediction, it becomes challenging to trust the accuracy and reliability of these algorithms [74]. The absence of transparency and explicability surrounding these AI algorithms may present a substantial impediment to the broad-scale implementation of AI-assisted selection approaches within sheep breeding programs, as producers may be reluctant to depend on models that they are unable to fully comprehend. Therefore, it is crucial to integrate quantitative insights derived from data with nuanced qualitative observations of behavior, such as experienced stockmanship, to holistically capture the multifaceted aspects of maternal care and ensure responsible implementation of AI-driven tools.

## 5. Proposed Model: ReproBehaviorNet—An AI-Driven Selection Framework for Reproductive Traits in Sheep

Drawing on the insights presented in this review, the authors propose an integrated, AI-driven selection framework, ReproBehaviorNet, specifically designed to capture and utilize behavioral indicators linked to reproductive success in sheep. It should be noted that ReproBehaviorNet is a conceptual framework introduced in this review and is not a commercially manufactured or available product. While AI tools are increasingly being used to monitor estrus, lambing behavior, and maternal traits, a unified model that translates this information into selection-based decision-making has yet to be introduced. ReproBehaviorNet aims to fill this gap by offering a structured, modular selection model that links behaviorally derived data streams to genetic improvement strategies. ReproBehaviorNet is a multi-layered decision support system that combines continuous behavioral monitoring, physiological sensing, and ML-based prediction to identify and select individuals with superior reproductive potential. The model focuses on behavioral phenotypes, traits that are observable, non-invasive, and indicative of key reproductive stages such as age at puberty, estrus, mating performance, maternal bonding, and lambing ease. The mapping of reproductive behaviors to biological processes and the AI applications used in the ReproBehaviorNet model are shown in Table 3.

These data are continuously collected and synchronized through a sensor cloud environment integrated with a behavioral annotation platform.

**Figure 1:**
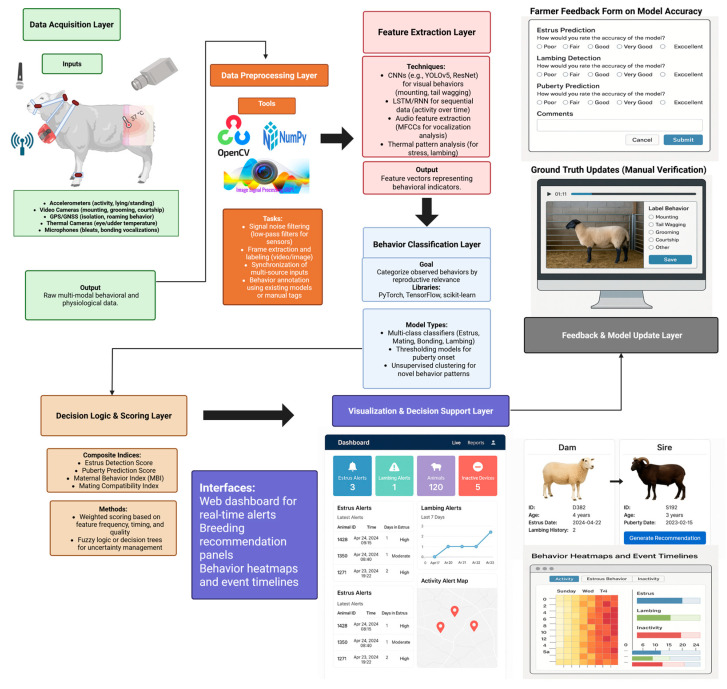
ReproBehaviorNet: an AI pipeline designed for the automated identification and scoring of reproductive behaviors in sheep.

The identification and scoring of reproductive behaviors in livestock, particularly in sheep, requires a robust and scalable AI infrastructure. The ReproBehaviorNet architecture (Figure 1) illustrates the full computational pipeline, including data acquisition, feature engineering, behavior classification, decision logic, and model retraining via feedback loops and transfer learning. This pipeline is designed to capture and analyze behavioral indicators of reproductive events—such as estrus, parturition, and maternal care—using sensor technologies and ML algorithms, with feedback loops for continuous improvement and adaptation across different breeds and production environments. The system architecture consists of sequential layers encompassing sensor-based data acquisition, preprocessing, feature extraction, behavior classification, scoring and inference, visualization, and model updating. Each layer plays a specific role in transforming raw data into meaningful indicators of reproductive status, ultimately enhancing selection accuracy and reproductive efficiency through precision livestock farming tools. While the model brings together validated technological components, ReproBehaviorNet remains a conceptual framework and has not yet been fully implemented or benchmarked against traditional selection methods. This represents a current limitation and offers a valuable direction for future research.

The architecture comprises seven functional layers: (1) The Data Acquisition Layer collects multi-modal behavioral and physiological data using accelerometers, video/audio, GPS, and thermal imaging sensors. (2) The Preprocessing Layer performs noise filtering, temporal synchronization, and annotation using platforms such as OpenCV and NumPy. (3) The Feature Extraction Layer applies Convolutional and Recurrent Neural Networks (e.g., CNNs, LSTM) and thermal/audio analysis to extract structured behavioral features. (4) In the Behavior Classification Layer, machine learning algorithms (e.g., PyTorch v1.13.0, scikit-learn v1.2.2) are employed to classify behaviors associated with estrus, lambing, puberty onset, and maternal care. (5) The Decision Logic & Scoring Layer integrates behavioral outputs into composite indices (e.g., Estrus Detection Score, Maternal Behavior Index) using weighted scoring and fuzzy logic. (6) The Visualization & Decision Support Layer delivers actionable insights through a user-friendly dashboard, offering real-time alerts, heatmaps, and breeding recommendations. Finally, (7) the Feedback & Model Update Layer incorporates manual ground truth verification and farmer feedback through structured forms, enabling model refinement via online learning and transfer learning to ensure adaptability to novel herds, breeds, and management systems.

## 6. Conclusions

This review underscores the growing potential of artificial intelligence as a transformative tool in sheep breeding, particularly for the selection of reproductive traits based on behavioral indicators. By integrating non-invasive technologies such as wearable sensors, video tracking, and ML algorithms, breeders can identify key behaviors—such as estrus expression, mating performance, and maternal care—with greater accuracy and in real time. The proposed ReproBehaviorNet model illustrates how behavioral data can be mapped to biological functions and AI processes, offering a scalable and practical framework for decision-making in both intensive and semi-intensive production systems.

These advances allow for the early identification of high-performing animals, reduced labor demands, and more welfare-friendly breeding programs. However, for responsible and sustainable adoption, several factors must be considered. Ethical and transparent use of algorithms is essential to avoid biases, ensure data privacy, and maintain trust in predictive models. Moreover, addressing implementation costs, user training, and technological infrastructure is vital to support producers, especially those operating in low-input systems. The development of user-friendly platforms, capacity-building initiatives, and participatory technology transfer programs will be key to ensuring inclusive access and the successful integration of AI tools on farms.

By bridging behavioral science and livestock technology, AI has the potential to significantly enhance reproductive efficiency, sustainability, and welfare outcomes in modern sheep farming, provided that it is implemented with responsibility, accessibility, and a producer-oriented design.

## Figures and Tables

**Table 1:** A summary of the sensor technologies used in sheep research relevant to reproductive monitoring.

Sensor Type	Most Common Use	Applications	Attachment	References	Relation to Reproductive Behavior
GPS	Tracking movement and location	Behavior, environment, health, method validation	Collars, harnesses	[41,42,43]	GPS can be used to monitor ewe movement near lambing, mating behavior
Motion Sensors (Accelerometers, IMUs)	Posture, walking, activity	Sensor validation, behavior	Collars, leg bands, harnesses	[44,45,46]	Motion sensors can detect restlessness, mounting, lambing signs
Heart Rate Monitors (HRM)	Physiological monitoring (stress, welfare)	Health, welfare	Collars, chest straps	[47,48,49]	HRM can be used to assess physiological stress and responses during estrus and parturition
Jaw/Bite Sensors	Feeding behavior	Feeding behavior	Jaw-mounted	[50,51,52]	These sensors can be linked to feeding behavior changes around lambing and mating
Contact Loggers	Social/contact behavior	Behavior, sensor validation	Collars, ear tags	[53,54,55]	Loggers can detect proximity and contact during mating and ewe–lamb bonding
Other Sensors	Various (temperature, respiration, etc.)	Diverse experimental focus	Custom methods (horns, fleece, etc.)	[56,57,58]	Limited used in specific studies (e.g., disease, respiration, temperature)

**Table 2:** Non-invasive digital tools for detecting reproductive traits and behavioral indicators in sheep.

Category	Application Area	Sensor Types	Model Types	Performance Metrics	Cases of Use in Sheep
Facial Recognition	Individual identification, breed classification, emotional state (pain/fear)	High-resolution RGB cameras, thermal infrared cameras	CNN, VGG variants, YOLOv8n, facial landmark detection networks	Up to 96.1% accuracy in pain/fear expression detection; YOLO-based breed ID > 95%	Monitoring pain post-procedure (e.g., tail docking), breed differentiation, individual tracking without tags
Body Metrics Measurement	Liveweight estimation, body condition scoring (BCS), growth monitoring	Depth cameras, 3D imaging systems, image processing with stereo vision	Computer vision algorithms, regression-based CNNs	Accurate within ±2–3 kg for bodyweight in penned settings; BCS prediction R^2^ > 0.9	Automated weighing without handling, early detection of poor body condition in extensive systems
Behavioral Monitoring	Detection of feeding, walking, standing, mounting (estrus), lambing events	Wearable accelerometers (ear, leg, jaw), RGB video cameras, microphones	YOLOv5, CNN-RNN hybrid networks, LSTM, rule-based activity classifiers	Estrus detection mAP > 99% with YOLOv5; lambing recall up to 0.94	Estrus detection for timed AI, predicting lambing time, identifying abnormal locomotion
Physiological Monitoring	Temperature mapping, respiratory rate, heart rate, pregnancy detection	Thermal cameras (eye, udder), piezoelectric belts, handheld ultrasound	Segmentation-based CNNs, object detection models, anomaly detectors	Thermal-based respiratory rate detection error < 2 breaths/min; pregnancy ultrasound > 90% accuracy	Non-contact fever screening, early disease or heat stress detection, automated pregnancy scanning

Abbreviations: CNN = Convolutional Neural Network; VGG = Visual Geometry Group; YOLO = You Only Look Once (object detection algorithm); LSTM = Long Short-Term Memory; mAP = mean Average Precision; R^2^ = Coefficient of Determination; AI = artificial insemination; BCS = body condition score; RGB = Red Green Blue (standard color image); RNN = Recurrent Neural Network.

**Table 3:** AI-assisted mapping of reproductive behaviors in sheep for breeding optimization.

Age Group	Target Trait	Observed Behavior	Sensor Type	AI Processing	Selection Relevance
Prepubertal ewe lambs	Puberty prediction in ewe lambs	Increased locomotion, vocalization, early mounting attempts	Accelerometers, video	LSTM for temporal trends; YOLOv5 for behavior tagging	Early identification of replacement breeders
Prepubertal ram lambs	Sexual maturity estimation	Flehmen response, sniffing, mounting, nudging	Video, thermal camera	CNN for visual traits; rule-based scoring for sexual behavior onset	Selection of early-maturing rams for artificial insemination centers or natural mating
Adult females	Estrus and lambing	Tail wagging, mounting by others, isolation, pawing ground	Accelerometers, GPS, video	Hybrid CNN-RNN model for stage prediction	Accurate timing for artificial insemination or mating, measuring estrus synchronization efficiency, predictive of lambing event, dystocia management
Adult males	Mating performance	Courtship intensity, ejaculation latency, hierarchy behaviors	Proximity loggers, video	Social interaction mapping; behavior–frequency analysis	Sire fertility prediction, mating compatibility
Postpartum ewes	Maternal bonding	Grooming, nursing initiation, proximity to lambs	Microphones, proximity sensors, video	Maternal Behavior Index (MBI) calculation based on behavior-scoring models	Lamb survival, dam selection, maternal instinct evaluation

Abbreviations: YOLO = You Only Look Once (object detection algorithm); LSTM = Long Short-Term Memory; CNN = Convolutional Neural Network; RNN = Recurrent Neural Network; GPS: Global Positioning System.

## Data Availability

The data supporting the reported results are available on request from the corresponding author. The data are not publicly available due to privacy and ethical restrictions.

## Abbreviations

The following abbreviations are used in this manuscript:

AI	Artificial intelligence
ML	Machine learning
PLF	Precision livestock farming
CNN	Convolutional Neural Network
RNN	Recurrent Neural Network
LSTM	Long Short-Term Memory
YOLO	You Only Look Once
MBI	Maternal Behavior Index
GPS	Global Positioning System
GNSS	Global Navigation Satellite System
IMU	Inertial measurement unit
RGB	Red Green Blue (color model)
BCS	Body condition score
HRM	Heart rate monitor
